# Diversity of P1 phage-like elements in multidrug resistant *Escherichia coli*

**DOI:** 10.1038/s41598-019-54895-4

**Published:** 2019-12-11

**Authors:** Carola Venturini, Tiziana Zingali, Ethan R. Wyrsch, Bethany Bowring, Jonathan Iredell, Sally R. Partridge, Steven P. Djordjevic

**Affiliations:** 1 Centre for Infectious Diseases and Microbiology, The Westmead Institute for Medical Research, The University of Sydney and Westmead Hospital, Sydney, NSW Australia; 2 The ithree Institute, University of Technology Sydney, Sydney, NSW Australia

**Keywords:** Bacteriophages, Antimicrobial resistance

## Abstract

The spread of multidrug resistance via mobile genetic elements is a major clinical and veterinary concern. Pathogenic *Escherichia coli* harbour antibiotic resistance and virulence genes mainly on plasmids, but also bacteriophages and hybrid phage-like plasmids. In this study, the genomes of three *E. coli* phage-like plasmids, pJIE250-3 from a human *E. coli* clinical isolate, pSvP1 from a porcine ETEC O157 isolate, and pTZ20_1P from a porcine commensal *E. coli*, were sequenced (PacBio RSII), annotated and compared. All three elements are coliphage P1 variants, each with unique adaptations. pJIE250-3 is a P1-derivative that has lost lytic functions and contains no accessory genes. In pTZ20_1P and pSvP1, a core P1-like genome is associated with insertion sequence-mediated acquisition of plasmid modules encoding multidrug resistance and virulence, respectively. The transfer ability of pTZ20_1P, carrying antibiotic resistance markers, was also tested and, although this element was not able to transfer by conjugation, it was able to lysogenize a commensal *E. coli* strain with consequent transfer of resistance. The incidence of P1-like plasmids (~7%) in our *E. coli* collections correlated well with that in public databases. This study highlights the need to investigate the contribution of phage-like plasmids to the successful spread of antibiotic resistant pathotypes.

## Introduction

*Escherichia coli* is a ubiquitous Gram-negative rod commonly found as a commensal inhabitant of the mammalian gut, but also capable of causing a wide range of diseases (from diarrhea to urinary-tract infections, meningitis and sepsis) in both humans and animals^1^. The global spread of multidrug resistance (MDR) in pathogenic *E. coli* limits treatment options, imposing a significant burden on health and veterinary agencies worldwide^2^. Successful dissemination of *E. coli* pathotypes depends on the acquisition and carriage of niche-specific traits that allow for stable colonization and persistence^1,3^. The genes encoding such traits are often associated with mobile genetic elements (MGEs), such as plasmids and bacteriophages^4–6^.

In *E. coli*, MDR spreads predominantly through horizontal gene transfer of antimicrobial resistance genes (ARGs), residing on large self-mobilizable plasmids and often co-localized in large complex resistance loci with transposons and insertion sequences (IS)^6,7^. Virulence genes can also be found on the same plasmids as ARGs^8,9^. A crucial evolutionary step in the emergence of highly virulent MDR *E. coli* clones, a cause of major disease outbreaks worldwide, was the acquisition of MDR plasmids^10–13^. However, other elements of the mobile genome, such as bacteriophages, also play a role in the dissemination of these adaptive traits^5,14,15^, and a better understanding of their diversity and of the extent of their contribution to pathotype adaptation is critical^16,17^.

**Figure 1:**
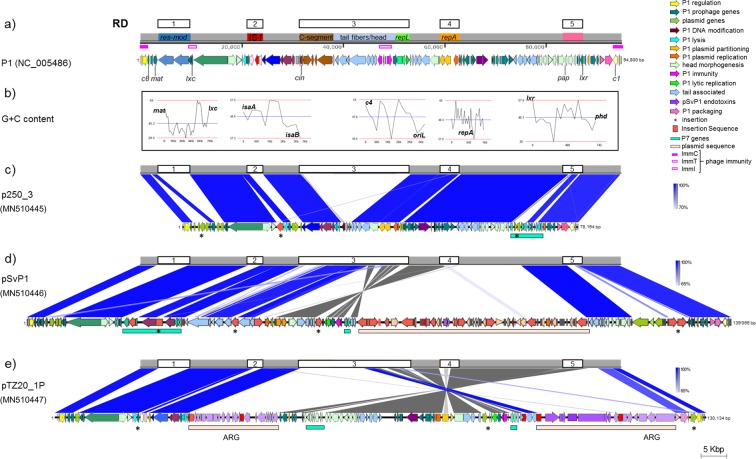
Comparison of P1-like genomes with coliphage P1 (NC_005856). (**a**) P1 genome mod749::IS*5* c1.100 thermosensitive mutant^19^. RD, regions of difference from P1 genome in P1variants. (**b)** Low G + C content regions in the P1 backbone associated with recombination hot-spots (RD1-5). (**c)** pJIE250-3, from clinical human *E. coli* ST405. The region between the P1-like operons for ‘head processing’ (*prt*, *pro*) and ‘lysis’ (*lydE*, *lydD*, *lyz*) includes insertion of IS*609* (positions 18,574–19,913; cleavage sites: left, TTAT; right, TCAA). (**d)** pSvP1, from porcine ETEC, with IS-mediated rearrangements leading to acquisition of F-type ETEC plasmid sequence including enterotoxin genes. From the end of *ssb* to the start of the ‘C segment’ (18,833–30,870), pSvP1 is closely related to P7 (96% overall nucleotide identity) with the addition of an IS*10*-like element (20,058–21,386) and IS*609*-like sequence (25,544–26,881). (**e)** pTZ20_1P, from commensal porcine *E. coli*, with plasmid fragments carrying antibiotic resistance genes (ARG). IS-mediated rearrangements led to the inversion of the P1 segment between *tub* and *lpa*, and deletion of P1-associated orfs (~11 kb encompassing the C-segment, tail fibers, ‘base plate and tail tube’ modules). The intergenic region between position 48,153 and 49,085 has 99% nucleotide identity to that of recently sequenced P1variants, but not P1 itself, and closer identity to P7 than P1 prevails in the ‘plasmid replication’, ‘lytic replication’ and ‘antirepressor’ operons. Between positions 89,842–89,967, corresponding to the P1 *ant1/2* overlapping orfs (immunity determinants), there is a small 126-bp gap. Schematics of genome sequences generated using SnapGene Viewer 4.1.4 (GSL Biotech; available at snapgene.com), where colors of coding sequences indicate different functional modules. Genome comparisons generated using Easyfig visualization tool^53^. Blue/grey blocks between P1 and each of the three P1-like plasmids schematics represent regions of conserved synteny with varying pairwise nucleotide identity according to BLASTn [scale bars for direct matches from 100% (dark blue) to 65% (light blue); grey indicates matches in reverse orientation].

Improvements in sequencing technologies, particularly availability of long-read sequencing platforms, have allowed the identification of numerous elements combining bacteriophage and plasmid modules in enterobacteria^15,18^. Many of these phage-like plasmids are derivatives of phages P1 or P7, closely-related temperate coliphages of the family *Myoviridae*^19^. These prophages infect a wide range of enteric Gram-negative bacteria, including *E. coli*, and are unusual in being commonly found as free circular plasmids stably maintained in bacterial cells without integration in the chromosome. Based on the presence of a plasmid replicon and plasmid maintenance genes, these elements were classified as belonging to plasmid incompatibility group (Inc) Y^19–21^ (Fig. 1a). Their stability and versatility is enhanced by the presence of a complex immunity circuitry encoded by three loci (ImmI, ImmC and ImmT^22^) and of a variable ‘C-segment’ in the tail fiber-encoding operon^19^. The immunity functions provide protection from infection by foreign DNA and competing phages; the C-segment works like a plasmid shufflon, inverted by a site-specific recombinase (Cin in P1, located 5′ to the C-segment), which confers tail fiber variability and, consequently, flexible phage host range^19,23^ (Fig. 1a).

The unique lifestyle of IncY phage-like plasmids facilitates transduction events^24^, and, in the era of multidrug resistant virulent clones, these elements may play an as-yet-underestimated role in the transfer of resistance and virulence genes. The genomes of parents P1 and P7 contain signature mobile elements, e.g. IS*1* in P1; IS*903* and Tn*2* with *bla*_TEM-1b_ (ampicillin resistance) in P7, and phage-like plasmids carrying ARGs linked to transposable elements have been sequenced from enteric bacteria isolated worldwide. P1/P7-like elements carrying extended spectrum β-lactamase genes have been identified in human clinical *E. coli* (e.g. *bla*_SHV-2_^25^) and *Klebsiella pneumoniae* strains (e.g. *bla*_CTX-M-15_^26^), and in *Salmonella* from pigs (*bla*_CTX-M-27_^27^), and carrying colistin resistance (*mcr-1*) in *E. coli* from humans^18^ and animals^28,29^, pointing at clinical and livestock reservoirs.

Here, we have carried out detailed comparisons of the genomes of three P1-like elements from Australian clinical and veterinary *E. coli* collections to identify genomic traits that may indicate the adaptive capacities and ease of spread/persistence of these elements: pJIE250-3, with no accessory genes, from an MDR *E. coli* isolated from a hospitalized human, and pTZ20_1P carrying ARGs, and pSvP1 carrying ETEC virulence genes, both from porcine *E. coli*.

## Results

### Genomes of P1-like plasmids

For all three phage-like elements PacBio sequencing and assembly generated one contig. *E. coli* TZ20_1P does not carry any plasmids other than pTZ20_1P, while JIE250, host of pJIE250-3, carries an MDR F-type plasmid^30^ and SvETEC, host of pSvP1, carries MDR IncHI2 and IncI1 plasmids^31,32^ and an FII: (F10:A-:B)-type plasmid encoding additional virulence factors and tetracycline resistance^32^. pJIE250-3, pSvP1 and pTZ20_1P are all variants of coliphage P1 (Fig. 1; Table 1), although some operons in each genome have closer identity to P7 (GenBank AF503408; Fig. 1c–e). pJIE250-3 appears to be a P1 derivative that has lost lytic replication functions and does not contain any cargo genes. In pSvP1 and pTZ20_1P, the P1-like backbone is interrupted by different IS-mediated insertions of plasmid fragments: a large section from an ETEC F-type plasmid carrying virulence determinants in the former, and plasmid backbone plus modules carrying ARGs in the latter.

**Table 1:** Main non-P1 features in phage-like plasmids pJIE250-3, pSvP1 and pTZ20_1P^*^.

	RD-1	RD-2	RD-3	RD-4	RD-5
**P1 NC_005486** (94,800 bp)	3,469–9,749 *res*-*mod*(IS5)	21,540–24,243 *isaA-*IS*1-isaB*	31,659–53,453 *cin* - C-segment, tail fibers, baseplate and tail tube, ImmI, lytic replication *genes* - *blpB*	58,774–62,949 plasmid partition and replication (*parSAB*, *incA, repA, incC, oriR*)	83,787–86,837 *pap* - head processing *genes* - *lxr*
**pJIE250-3** (78,164 bp)	6,556–8,293 2 orfs including putative transcriptional regulator [16 *E. coli* MGEs, >98%]	21,420–23,805 2 orfs including putative phosphohydrolase [5 *E. coli* MGEs, >99.5%]	31,084–33,367 4 orfs: invertase gene [*complement*; aa 98.5% Min p15B X62121^61^]; tail fiber gene [95.2% p15B] plus 2 inverted repeats; 2 orfs including antibacterial toxin [*E. coli* MGEs 94–99%; no match on full length]	42,844–43,715 2 orfs [15 *E. coli* MGEs, >99.6%] substitute P1 sequence preceding *oriR* deleting P1 *upfA*	64,151–69,831 identity to P7 (≥98% nucleotide level with small gaps); approximately where IS*903* is in P7, 2 non-P7 orfs [*Salmonella* MGEs (CP034821; KU760857 - 96.1%] substitute *upf91.7*
**pSvP1** (139,066 bp)	3,476–7,042 7 orfs: 5 including homologs of P1 *icd-ant1/2* [STEC *E. coli* plasmids CP021340 and CP021336 91.8%] + 2 including a putative transcriptional regulator [*Klebiella variicola* and *Serratia liquefaciens* chromosomes 65.5%; aa id to *E. coli* proteins]	18,834–22,562 2 orfs: P7 *nmpC* interrupted by IS*10* and P7 *odaA* [P7 AF503408, 97.9%]	30,866–44,921 + 56,523–65,283 partial invertase orf [N-catalytic domain of the P1 Cin Ser-recombinase]; *sit* interrupted by IS*2*; IS*Ec84*-mediated inversion of backbone *pmgB to blpB*: *pmgB* interrupted by IS*Ec84;* IS*186B* insertion with deletion of *rlfA*, *rlfB* (lytic replication) and *pmgF* (putative morphogenetic function) substituted by 2 orfs including antibacterial toxin [*E. coli* MGEs >99%]	48,004–49,273 2 orfs [*E. coli* MGEs, >98%] inserted between *repA* and *parA*	119,363–126,907 6 orfs including type I restriction modification, DNA methyltransferase substituting *pmg* genes (putative morphogenetic function) [*E. coli* IncY MDR plasmid pR15_MCR-1 MK256965.1, 95%]
**pTZ20_1P** (130,134 bp)	3,468–4,981 2 orfs including a putative phosphokinase and partial P1-*lxc* homolog [14 *E. coli* MGEs >99%]	42,873–43,704 2 orfs unknown function: 1, interrupts *isaA*; 2, replaces *isaB*	26,194–26,583 + 84,696–96,450 IS*26*-mediated rearrangements and deletions: *Δcin*, deleted C-segment-tail fibers-baseplate-tail tube, *Δtub*, *Δsit*; *sit-blpB* inverted. 3 orfs between *repL* and *blpB* substitute *rlfA*-*rlfB*-*pmgF* [*E. coli* MGEs CP010146, 99.8%]	75,879–79,888 *parAB* 99.8% P7 (minimal similarity to P1); incompatibility regions limited identity to P1/P7 [P7: 5′ *parA*, 91.6%; 3′ *parB* 81%]	48,154–54,564 8 orfs (*pmgU-pap*) putative morphogenic function <40% P1/P7 [*E. coli* MGE CP035333, 99.4%]

^*^Throughout: [x,%], best BLASTn matches in the GenBank database (nucleotide percentage identity over 100% length if not otherwise stated); aa, BLASTp.

#### Common regions of difference (RD)

Comparative analysis with the chosen P1 reference genome^19^ (P1 mod749::IS*5* c1.100 mutant, GenBank NC_005856) revealed five hot-spots of sequence variation common to all three elements (RD-1 to RD-5; Fig. 1a), mainly associated with boundaries between regions of average G+C content and AT-rich loci (Fig. 1b). However, in each RD, changes from the P1 backbone were unique for each element (Fig. 1c–e; Table 1). The *res* and *mod* genes (restriction modification function; RD-1) and IS*1* of P1 (RD-2) are missing from all three elements, replaced by orfs unique to each, but generally highly similar to sequences identified in other phage-like plasmids from *E. coli* (Table 1). Between RD-1 and RD-2, the ‘C1 modulation’ (implicated in control of lysogeny), ‘antirestriction’, ‘head processing’, ‘viral architecture’, and ‘lysis’ operons largely match those of P1 or P7^19^ (Fig. 1c–e), except for IS*609* inserted in pJIE250-3 and pSvP1 (Fig. 1c,d; Table 1). RD-3 corresponds to the P1 C-segment and adjacent genes, encoding tail structures (fibers, baseplate, tail tube), the ImmI-associated region (immunity function: *sim* operon, *icd*, *ant1/*2;), *kilA* and the ‘lytic replication’ module (*repL*, *oriL*, *rlfA* and *B*)^19,22,33^ (Fig. 1a). pJIE250-3 has only one tail fiber gene and all other P1 modules are missing (Fig. 1c). In pSvP1 and pTZ20_1P, RD-3 includes IS-mediated insertions of plasmid backbone fragments and associated resistance or virulence genes, with consequent deletion of various segments of the P1 backbone (Fig. 1d,e; Table 1). A gene related, but not identical, to *cin* of P1, encoding the invertase responsible for tail switching, was identified in each element (Table 1). However, this gene was truncated (pSvP1; pTZ20_1P) or in reverse orientation (pJIE250-3), when compared to P1-*cin*, and/or its target (C-segment) was greatly modified (pJIE250-3 and pTZ20_1P), likely impairing functionality of the switch (Table 1).

RD-4 corresponds to the P1 plasmid replicon (including *parAB* and *repA*) and flanking regions (Fig. 1). pJIE250-3 is almost identical to P1 in this region except for a short insertion 5′ of *oriR*, containing two orfs. In both pSvP1 and pTZ20_1P, the main changes here involve sequences encoding incompatibility determinants, with potential modification of replicon function (Table 1). RD-5 is located between *pap* and *lxr*, where in all three elements some of the genes encoding head morphogenesis in P1 are modified (closer identity to P7) or deleted (Fig. 1; Table 1). Additionally, in pTZ20_1P two short hypothetical orfs and a gene encoding an antirepressor (*ant*) are found inserted in the P1 backbone between *pacB* and *c1*, as in other phage-like plasmids (e.g. RCS47, NC_042128; Fig. 1e).

#### Unique features of P1-like plasmids

##### pJIE250-3

In pJIE250-3, five additional orfs are inserted between the *c8* and *mat* genes at an AT-rich junction, near a cluster of 4× 4-bp direct repeats (DR) (ATTG; 1,957–1,982), close to the insertion site of Tn*2* in P7. A similar segment was found in nine other *E. coli* plasmids (e.g. CP032890.1, CP019262.1 and MG825383.1, best matches with >99.5% nt identity) and in the chromosome of *E. coli* ST2747^34^ (CP007393.1, >99.4% nt identity), and includes genes encoding a putative transcriptional regulator of metabolic functions as well as a putative resolvase.

##### *Plasmid sequence in pSvP1*

**Figure 2:**
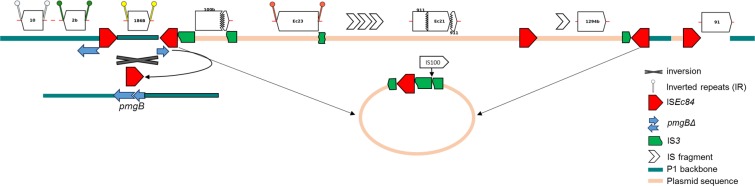
Insertion sequences and IS-mediated rearrangements in the pSvP1 genome. IS*10* (IR_R_, 21,385–21,336, and IR_L_, 20,057–20,106; 9-bp DR, TGCTCTGCA) interrupts the P7 porin precursor gene *nmpC*, and at 40,334 IS*2* interrupts the orf related to P1 *sit* (5-bp direct repeats, ACCAA). IS*186B* (IS*4* family; 10-bp direct repeats, GGATCTCTCC) is inserted between *bplB* and the ‘lytic replication’ module, causing deletion of P1-like sequence associated with lytic replication (*rlfA*, *rlfB*) and with putative morphogenetic function (*pmgF*). IS*Ec84.1* interrupts a P1 *pmgB* homolog, but the remaining part of *pmgB* lies adjacent to IS*Ec84.2* in the opposite orientation. As shown, reversing the segment between IS*Ec84.1* and IS*Ec84.2* and removing an IS*Ec84* would regenerate a complete *pmgB* gene. IS*Ec84* insertion mediated acquisition of a large fragment of ETEC plasmid containing multiple IS elements. The region between IS*Ec84.2* and IS*Ec84.4* corresponds to ETEC plasmid sequence containing multiple IS elements. IS*3* fragments (green) flanking these IS*Ec84* suggest insertion of a circular molecule carrying IS*Ec84* inserted in IS*3* (as shown) by recombination in a copy of IS*Ec84* in the P1-like backbone. IS*Ec84* and IS*91* may also have been responsible for the acquisition of a region related to *E. coli* IncY MDR plasmid pR15_MCR-1 (95% nucleotide identity; GenBank MK256965.1), containing CDSs involved in restriction modification (type I) and DNA methylation with deletion of several P1 *pmg* genes (putative morphogenetic function). Schematic not to scale.

Five copies of IS*Ec84* (IS*Ec84.1*- IS*Ec84.5*), a member of the IS*91*-family (rolling circle transposition)^6,35^, are present in RD-3 of pSvP1 and rearrangements mediated by IS*Ec84* seem to have been largely responsible for modification of the P1 backbone here (Fig. 1d; Fig. 2). The P1-like backbone (*pmgB* to *upfA*) between oppositely-oriented IS*Ec84.1* and IS*Ec84.2* is inverted, presumably as a result of IS-mediated recombination (Fig. 2). IS*Ec84.2* is followed by a fragment of the left end (inverted repeat, IR_L_) of IS*3* interrupted by IS*100*, then a region that includes several IS (complete and partial) within a large segment (~41.5 kb; at position 67,105) matching F-type plasmids of porcine ETEC [e.g. p14ODTX (MG904993.1; 94,167 bp); pUMNK88_Ent (CP002732.1; 81,475 bp)^36^] (Fig. 1d). Addition of this plasmid backbone segment has led to acquisition of a new plasmid replicon (*rep*FIB), endotoxin genes (*stb*, encoding the heat stable enterotoxin II, and *eltAB*, encoding the heat labile enterotoxin), and a toxin-antitoxin stability system. Several other IS, including IS*Ec84*.3, are found within this insertion exactly as in the ETEC plasmids (Fig. 2). The acquired plasmid sequence ends at IS*Ec84.4*, flanking another partial IS*3*. This arrangement suggests incorporation of a plasmid-derived circular molecule containing IS*Ec84* within IS*3* into a P1-like backbone (Fig. 2) and concomitant deletion of the P1 DNA methylation operon, phage tRNAs, DNA helicase and part of the ‘baseplate/tail tube’ module (between *upfA* and *24*; Fig. 1a,d).

##### *Plasmid sequence in pTZ20_1P*

**Figure 3:**
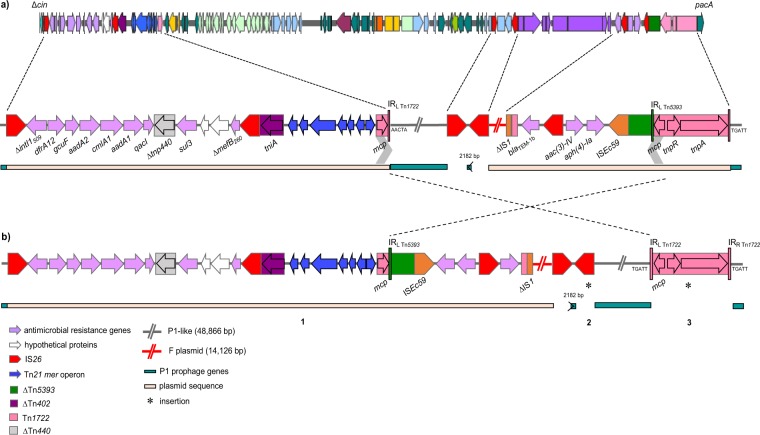
Multiple antibiotic resistance regions and rearrangements in pTZ20_1P. (**a)** Schematic of the complete pTZ20_1P genome showing insertion points of plasmid regions containing resistance genes, transposons and IS. Two oppositely-oriented copies of part of Tn*1722* (marked by grey chevrons) are found 79,484 bp apart and the 5-bp immediately adjacent to the IR_L_ and IR_R_ of Tn*1722* are reverse complements of one another (AACTA; TGATT). (**b)** Reversing the region between the Tn*1722* fragments (to mimic homologous recombination) regenerates a complete Tn*1722*, with matching 5-bp direct repeats (TGATT) marking the insertion. Similarly, reversing a 2,182 bp segment between two inversely-oriented copies of IS26 (to mimic intramolecular transposition by IS*26*^60^) results in an IS*26* flanked by matching 8-bp direct repeats. This, presumably ancestral, version of pTZ20_1P corresponds to a P1-like backbone with three separate insertions (1) an 39,980 bp region bounded by directly oriented copies of IS*26*, (2) IS*26*, and (3) Tn*1722*. Almost identical sequence after the leftmost IS*26* through the *sul3*-type integron to IR_L_ of Tn*21* (1) is found in a few *Salmonella* ssp. and *E. coli* plasmids in INSDC databases. Tn*21* truncates Tn*1722*, which is also truncated by the IR_R_ end of Tn*5393*, with novel boundaries in both cases. Tn*5393* is truncated by IS*Ec59*, an IS*6* family element, with another copy of IS*26* flanking a 2,447 bp region containing the *aph(4)-Ia* and *aac(3)-IV* genes. The IR_R_ end of IS*26* truncates the IR_L_ end of Tn*2*, leaving an intact *bla*_TEM-1b_ gene. IR_R_ of Tn*2* is immediately followed by 49 bp of Tn*5393* IR_L_, 106 bp of the IR_L_ end of Tn*1722* and 177 bp of the IR_L_ end of IS*1*. The 14,126 bp region between IS*1Δ* and the next IS*26* corresponds to *finO-traX-traI-traD-traT-traG-traHΔ* genes matching (~98% identity) several F-type plasmids in INSDC, some of which have the same boundary with IS*1*. Diagrams not to scale.

pTZ20_1P contains a *sul3* type class 1 integron in which *intI1* is truncated by IS*26* (Δ*intI1*_509_) and the cassette array is *dfrA12-gcuF-aadA2-cmlA-aadA1-qacI*, conferring resistance to trimethoprim, streptomycin and spectinomycin, chloramphenicol and quaternary ammonium compounds. Beyond *sul3* (sulphonamide resistance), the *mefB* gene is truncated by IS*26* (Δ*mefB*_260_). Another resistance region includes *bla*_TEM-1b_ (ampicillin resistance), *aac(*3*)-IV* (gentamicin and tobramycin resistance) and *aph(4)-Ia* (hygromycin resistance) genes associated with complete and truncated IS and fragments of various Tn*3*-family transposons (Fig. 3). This set of resistance genes fully accounts for the antibiotic resistance phenotype of TZ20_1P.

No DR indicative of insertion were identified adjacent to any mobile elements. However, sequences of the expected DR length that are reverse complements of one another are found adjacent to the terminal IR of two Tn*1722* fragments (5 bp) and two different copies of IS*26* (8 bp). This suggests inversions, consistent with the comparison between pTZ20_1P and P1 (Fig. 3). Reversing these generates a (presumably ancestral) version of pTZ20_1P with three distinct insertions in the P1 backbone. One comprises a 39,980 bp region bookended by directly-oriented copies of IS*26* and containing the integron and other resistance genes, plus 14,126 bp matching part of the *tra* region of several F-type plasmids in INSDC databases (Fig. 3). This suggests IS*26*-mediated transfer of part of a plasmid to a P1-like backbone. The other two are separate insertions of IS*26* and of a complete Tn*1722* directly into the P1-like backbone, each flanked by DR. The truncated *cin* gene and the packase-encoding gene *pacA* (88% nt identity to P1) flank the plasmid insertion (Figs. 1c and 3).

#### pTZ20_1P self-transfer ability

Induction, lysogeny and conjugation ability were tested using pTZ20_1P, as this element carries resistance markers and no additional plasmids were detected in its host, simplifying selection strategies. We evaluated whether pTZ20_1P is inducible, treating exponential growth phase cultures with mitomycin C or UV light. To confirm the presence of the induced phage-like element in the filtered suspension, we performed PCR for the replication genes *repA* (plasmid) and *repL* (phage), and linking Tn*1722* (plasmid) and the *pacA* gene (phage) (Fig. S1). PCR amplification was successful in all induced cultures, except samples exposed to UV light for 20 s (shortest exposure time; Supplementary Fig. S1a). No amplicons were obtained from DNA extracted from an un-induced filtered suspension of *E. coli* TZ20_1P. We tested the ability of pTZ20_1P to lysogenize different commensal *E. coli* host strains. Lysogens were only detected for *E. coli* WH17, not for *E. coli* J53 or WGNB13. PCR amplification of marker genes and whole genome sequencing (WGS) of lysogens confirmed the acquisition of pTZ20_1P (Supplementary Fig. S1b). The capacity of pTZ20_1P to transfer by conjugation was also tested, but no transconjugants were obtained, as expected due the absence of a complete conjugative transfer operon in the inserted plasmid segment (Fig. 3).

#### Incidence of P1-like plasmids in Australian *E. coli*

*In silico* screening of available genome sequences for the P1-associated *repL* (phage) and *repA* (plasmid) replication genes, identified both in 8/117 (6.8%) isolates from a collection of porcine commensal *E. coli*, but only in 3/328 (1%) isolates from human clinical collections of MDR *E. coli* and *Klebsiella pneumoniae*. However, BLASTn searches of the INSDC database (accessed September 2018) using the same targets returned 54 entries containing both genes (*repA* >98% identity; *repL* >97% identity). Using the search term ‘plasmids Escherichia coli’ to query genome entries in the NCBI database, we found 1429 sequences annotated as ‘plasmids’. Of these, ~7% had characteristics indicative of phage sequences (e.g. in 97/1429 (6.7%) t-RNA presence), a frequency comparable to that in our local collections, and 22 of these were also identified by the BLASTn screening. Screening for pSvP1 in the genome of sister strain *E. coli* O157 734/3 showed the presence of this element in the Australian lineage since 1995^31^.

## Discussion

In the era of rising MDR, large conjugative plasmids have been recognized as the main vehicles of antibiotic resistance maintenance and transfer, particularly in Enterobacteria. Phages and phage-like plasmids, however, may also have a prominent role in the dissemination of accessory adaptive traits^5,15,37^. Enterobacteria phage P1 is known to infect and lysogenize *E. coli*, being maintained within the cell as an autonomous low-copy number plasmid^19^. It does not contain cargo genes of clinical interest and, being a prophage, has not been considered for therapeutic applications. Therefore, although P1 was discovered in *E. coli* over 50 years ago^19,22^, research efforts have mainly focussed on its properties as a molecular biology tool rather than as an active element of the accessory genome^22,38,39^. However, recent advances in sequencing technologies (e.g. PacBio) have facilitated the detection of numerous P1-like variants, though detailed characterization has been limited to a few carrying ARGs of clinical relevance^18,25,27,29^. In this study, we defined the fine diversity between three P1-like plasmids from *E. coli*, isolated from different reservoirs (animal and human), with the intention of identifying modifications that may associate with pathogen adaptation.

Analysis of pJIE250-3, pTZ20_1P and pSv1P revealed three different variants of P1 sharing common traits. pJIE250-3 is an example of a P1-derivative that has lost its lytic replicon, but retained P1 plasmid functions augmented by acquisition of additional plasmid-related orfs (e.g. transcriptional regulators). pTZ20_1P and pSv1P are genetic mosaics of P1-like elements and plasmid segments with ARGs and virulence determinants, respectively. The genomes of all three phage-like plasmids are defined by different features unique to each, and none has an exact match in INSDC databases. However, each genetic locus is shared with at least two other phage-like plasmids, highlighting the variability of phage-like MGEs, testament to their recognised transduction ability^19,39^. Whether this variability is a product of random reassortment due to generalized transduction or a product of the adaptive strategy of different pathotypes to their specialized environment is yet to be determined.

The variable regions (RD) distinguishing each element are associated with the same limited number of P1 genetic loci of low G+C content, where P1 genes previously described as recent acquisitions (e.g. *res-mod, sim, rfl*) are located^19^. These hot-spots tend to be related to host-range determinants (C-segment; tail fibers), replication functions (modification of lytic or plasmid replicons), and immunity encoding regions (ImmI specifically). Inspection of other P1/P7-like plasmids described in the literature confirms that these RD tend to be shared among elements isolated from different *E. coli* worldwide^18,25,28,29^. All three P1-like plasmids have a modified tail fiber operon when compared to P1, with an altered *cin* sequence, and lack a complete C-segment locus (tail tip switch^40^). Cin, a P1/P7 specific invertase, is responsible for the production of virions with different host specificity from the parent, a property presumably lost in these P1 variants, with possible consequent restriction of host range. In pJIE250-3, this feature is combined with loss of *repL* (essential for lytic replication), likely preventing self-mobilization of this element from its host, an *E. coli* clinical strain carrying multidrug resistance on a large F-type conjugative plasmid^30^. However, pJIE250-3 has retained P1 plasmid loci and acquired several additional genes with putative metabolic/DNA processing functions, which could allow for better stability in a fixed genomic background.

In P1, the integrity of the plasmid replicon (iterons), partitioning module and plasmid addiction systems, including *res-mod* (notably absent in the three variants), is important to ensure stable plasmid maintenance. The absence of *res-mod* may decrease protection from entry of foreign DNA, but there are indications that the additional genes acquired by the P1variants may provide functions facilitating adaptation to local environments (e.g. in pSvP1, acquisition of a type I restriction modification system, in pJIE250-3 transcriptional regulators etc.). In pSvP1, plasmid functions are also likely enhanced through acquisition of *rep*FIB and additional stability and maintenance modules in the inserted plasmid segment.

Negative impact on broad host-range could be associated with modification of the complex P1 immunity circuitry protecting the prophage by exclusion of both foreign phage and transducing DNA^19^. P1 has three immunity encoding regions, Imm I, T and C^22,33^. ImmC, with C1 repressor of lytic function, and ImmT, implicated in C1 modulation, promote plasmid (prophage) maintenance versus entry into the lytic cycle. ImmI (*sim, c4*, *icd*, *ant1/2*) has been ascribed a specific role in permitting P1 and P7 coexistence (with minor nucleotide differences between P1 and P7 responsible for their reported heteroimmunity) and adding flexibility to ImmC functions^33,41^. P1 and P7 are heteroimmune relatives known to readily recombine^42^, as evidenced in the genomes of our elements, but in pJIE250-3 and pSvP1, the ImmI region is modified, possibly compromising superinfection immunity functions. However, interestingly in pJIE250-3 the absence of ImmI is associated with the concomitant loss of *repL*, and in pSvP1 with the acquisition of homologs of P1 genes with putative immunity functions (*icd* and *ant1/2* in RD-1).

Here, we showed that P1-like elements in *E. coli* can co-exist with large plasmids carrying complex resistance regions, can pick up different cargo genes (MDR and virulence), and move into a different host. In the lysogenized commensal *E. coli* WH17, F-type replicons were detected and the MDR transferred as part of pTZ20_1P could potentially transfer to these. There have also been reports of transfer of non-conjugative plasmids between *E. coli* cells by transformation mediated by P1 phage lysis^43–45^, indicating that multiple modes of interaction and spread of these elements may shape the adaptive potential of a bacterial population^44^. Both pJIE250-3 and pSvP1 were found in the same cell as large plasmids and, provided that they are capable of productive lysis^45^ (less likely for pJIE250-3 lacking the *repL* gene), could promote or enhance plasmid movement by these mechanisms. As the mammalian gut is an excellent niche for ARG exchange via horizontal transfer, tracking these elements may become crucial in responding to the threat of rising MDR in enteric pathogens.

In pTZ20_1P, IS*26*-mediated transposition events likely led to the acquisition of the multidrug resistance region by the P1-like backbone, with IS*26* truncating both *intI1* (IS*26*-Δ*intI1*_509_) and *mefB* (IS*26*-*ΔmefB*_260_). The presence of an intact P_C_ promoter allows expression of the *dfrA12* gene in the cassette array, as confirmed by the strain’s antibiotic resistance phenotype (trimethoprim resistance), even though IS*26*-Δ*intI1*_509_ may prevent the integration of new gene cassettes into the array. The IS*26*-*ΔmefB*_260_ signature is frequently found on enterobacterial plasmids in combination with *sul3*, including in porcine and human *E. coli*^4,31,46^, while IS*26*-Δ*intI1*_509_ is much less common. IS*26*-*ΔmefB*_260_ and IS*26*-*ΔintI1*_509_ together represent a valid signature for tracking this specific class 1 integron in different hosts and environments.

The elements described here are examples of how P1 can evolve in different *E. coli* genomic backgrounds by modification of replication and host range properties in association with acquisition of cargo genes, showing that P1/P7-like plasmids may not only be capable of generalized transduction, but also be specific vehicles for spread and maintenance of virulence and antimicrobial resistance in both clinical and livestock production settings. MGEs coexisting within the same host interact with each other, affecting reciprocal stability, and their cooperative interactions should be considered when defining bacterial adaptive strategies.

## Methods

### Bacterial strains

Three *E. coli* isolates belonging to different sequence types (ST) were host to the three P1-like plasmids described here. *E. coli* TZ20_1P, ST372, phylogroup B2, serotype O6:H31, was isolated in January 2017 at the Elizabeth MacArthur Agricultural Institute (EMAI), Menangle, NSW, Australia, from faecal material from a healthy four-week-old piglet not previously treated with antimicrobials. *E. coli* SvETEC ST4245, phylogroup C, serotype O157, was also isolated from faecal material from a diarrhoeic piglet, in 2008^32^. *E. coli* JIE250, ST405, phylogroup B2, is a human isolate, part of a large clinical collection of *Enterobacteriaceae*^30^. This isolate was shown to carry a large conjugative F-type plasmid with a complex MDR region including multiple antibiotic resistance genes (e.g. *bla*_CTX-M-15_), transposons and IS^47^.

### PacBio sequencing, annotation and bioinformatic analysis of MGEs genomes

Genomic DNA was isolated and purified from bacterial cultures grown overnight using the Mo Bio Powersoil® DNA Isolation kit (Mo Bio, Carlsbad, CA, USA) or the DNAesy Blood and Tissue kit (Qiagen, Hilden, Germany), according to manufacturer’s instructions. Long-read sequencing was performed on the three *E. coli* isolates on a PacBio RSII Instrument at the Ramaciotti Centre for Genomics (UNSW, Sydney, Australia). Polishing and assembly of sequenced reads was performed at the Ramaciotti Centre using HGAP and CANU, and plasmids were closed using Circlator^48^. Errors in PacBio assemblies were checked and curated by alignment with Illumina short reads from whole genome sequencing of the *E. coli* hosts. P1-like genomes were first annotated using RAST*tk*^49^, then manually curated using BLAST functions^50^, SnapGene (GSL Biotech; available at snapgene.com) and Geneious v.9.1 (https://www.geneious.com). Plasmid virulence and resistance regions were also annotated using web-based software [Center for Genomic Epidemiology, www.genomicepidemiology.org; Galileo^TM^ AMR (formerly MARA), galileoamr.arcbio.com/mara/^51^]. All allelic variants of IS*26*^52^ detected in pTZ20_1P are referred to as IS*26* throughout. Comparisons with the reference P1 genome (P1 mod749::IS5 c1.100 mutant; GenBank NC_005486)^19^ were visualized using EasyFig.^53^.

### Transfer of pTZ20_1P

#### Phage induction and lysogenization of commensal *E. coli*

*E. coli* TZ20_1P was grown in lysogeny broth (LB; Becton Dickinson, Franklin Lakes, NJ, US), with vigorous shaking at 37 °C, to OD_600_ 0.3, and treated with mitomycin C at 0.05, 0.1, 0.15, or 0.2 μg/mL, or exposed to UV light for 20 s, 45 s or 1 min (UV Stratalinker 1800 (230 Vac, 2 A, 50 Hz), Stratagene, CA, US). Induced cultures were incubated at 37 °C for 3 h with gentle shaking and centrifuged at 4,000 x g for 20 min to remove bacterial cells debris. Supernatants were filtered and concentrated using 0.22 μm Amicon Ultra-15 filters (Sigma-Aldrich, St. Louis, MO, USA). Suspensions were stored overnight at 4 °C prior to lysogenization assays. Three previously characterised commensal *E. coli* strains (WGNB13 and WH17^54^ and J53^55^), which are streptomycin susceptible but carry other resistance genes suitable for counter-selection, were used as recipients to test the lysogenic ability of pTZ20_1P. Recipient strains grown to OD_600_ 0.6 in LB were pelleted by centrifugation and resuspended in LB supplemented with 1 mM CaCl_2_. Bacteria were mixed (1:1) with phage lysates, and the mix was incubated in LB broth (static conditions, 40 min) or on LB agar plates (overnight) at 37 °C. *E. coli* recipients were also mixed with un-induced *E. coli* TZ20_1P, as negative controls. The mixtures were plated on LB agar supplemented with Str 25 μg/mL (selection for phage-like plasmid) and incubated at 37 °C for 24 h and 48 h. All transfer experiments were performed in triplicate.

#### Characterization of lysogenic *E. coli*

To confirm that the suspension obtained after induction contained pTZ20_1P and its consequent stable acquisition by recipient strains, we performed PCR amplification of two regions: (1) the plasmid replication gene *repA* (repA-fw, AAAGCCGAGGGTTACGATGA, and repA-rev, ATGATACGGTTTTGCTCGCC; amplicon size 542 bp; this study), and (2) the phage lytic replication gene, *repL*, unique to the P1 component (RepL-fw and RepL-rev^25^; amplicon size 489 bp). To unequivocally confirm the identity of the transferred element, we also amplified the region linking plasmid (Tn*1722*) and phage (*pacA*) modules of this element (Tn1722, CAGACTGGAAGACGGGAAGT; *pacA* TCAGCCATTTCAGCCACAAC; amplicon size 428 bp; this study). Phage DNA was isolated from filtered induced suspensions by treatment with DNAse (10 mg/mL; 30 mins) and RNAse (100 mg/mL; 30 mins), followed by extraction and purification using the Wizard DNA Clean-up System/kit (Promega, Madison, WI, USA) following manufacturer’s instructions.

The genome of one representative *E. coli* lysogen (*E. coli* WH17 mitomycin C_0.2 μg/mL) was sequenced to confirm pTZ20_1P acquisition. Bacterial DNA was extracted and purified using the ISOLATE II genomic DNA kit (Bioline, London, UK). WGS was performed on the Illumina NextSeq (paired-end 150 bp × 2) platform at the Pathogen Genomics Unit, Centre for Infectious Disease and Microbiology – Public Health, Westmead Hospital (NSW, Australia). Briefly, total DNA concentration was quantified using Quant-it PicoGreen dsDNA Assay Kit (Invitrogen, Carlsbad, CA, USA) and 1 ng/µl of DNA was used to prepare DNA libraries using the Nextera XT Library Preparation Kit and Nextera XT v2 Indexes (Illumina, San Diego, CA, USA). Multiplexed libraries were sequenced using paired end 150 bp chemistry on the NextSeq. 500 NCS v2.0 (Illumina). Error rates were calculated using PhiX Sequencing Control v3 for each run. Demultiplexing and FastQC generation was performed automatically by BaseSpace (Illumina). Alignment between the lysogenic genome (raw reads) and the original pTZ20_1P PacBio sequence was generated and visualised using Bowtie2^56^ v2.3.0, SAMtools^57^ v1.4.1, and Tablet^58^ v1.19.05.28.

#### Conjugation assay

To assess the ability of pTZ20_1P to transfer via a plasmid-related mobilization mechanism, we performed conjugation assays using *E. coli* EC JM109 Rif^R^/Nal^R^, resistant to rifampicin (Rif) and nalidixic acid (Nal) as recipient^8^. A loopful each of donor (*E. coli* TZ20_1P) and recipient cultures were mixed thoroughly in saline (500 μL), and the mating mix was plated onto LB agar and incubated overnight at 37 °C. The mating lawn was resuspended in 1 mL saline and serial dilutions spotted in triplicate onto LB agar plates supplemented with Nal (30 μg/mL, Nal_30_ - recipient selection) and Nal_30_ plus ampicillin (100 μg/mL, Amp_100_) or streptomycin (25 μg/mL, Str_25_) to detect transconjugants (Nal_30_Amp_100_ or Nal_30_Str_25_, selection for pTZ20_1P), and LB only agar (total bacterial count). Donor and recipient were also separately plated on LB Nal_30_Amp_100_, LB Nal_30_Str_25_ and LB agar only as controls. Conjugation assays were also performed in the presence of *E. coli* HB101 containing the helper plasmid pRK600 with chloramphenicol resistance^8,59^. Donor and recipient, grown independently under the same conditions and plated onto LB supplemented with Str_25_ and Nal_30_ respectively, were used as controls.

### Occurrence of phage-like plasmids in Australian *E. coli* collections and NCBI databases

The occurrence of P1-like plasmids in available sequences was determined by querying the NCBI bacterial genomes database and by *in silico* BLAST-type searches with *repA* and *repL* sequences (minimum percentage identity 95%) of representative collections of local *E. coli* genomes.

## Supplementary information

Supplementary Figure 1

## Data Availability

The complete sequences of pTZ20_1P, pJIE250-3, and pSvP1 have been deposited in GenBank (NCBI) under accession numbers MN510447, MN510445, and MN510446 respectively. The complete raw read dataset for lysogenic *E. coli* WH17 mitomycin C_0.2 μg/mL containing pTZ20_1P has been deposited in the SRA (NCBI) database under Bioproject PRJNA565856 (SRR10127725). All other data generated or analysed during this study are included in this published article (and its Supplementary Information Files).
