# Supplementary material for: Diversity of P1 phage-like elements in multidrug resistant Escherichia coli
Source: Sci Rep. 2019 Dec 11;9:18861. doi: 10.1038/s41598-019-54895-4 (PMC6906374; doi:10.1038/s41598-019-54895-4)
Supplement: Supplementary file 1 — Supplementary Figure 1 [file 41598_2019_54895_MOESM1_ESM.docx]

**Supplementary Material**

**Diversity of P1 phage-like elements in multidrug resistant *Escherichia coli***

Carola Venturini^1,*,†^, Tiziana Zingali^2,*^, Ethan R. Wyrsch^2^, Bethany Bowring^1^, Jonathan Iredell^1^, Sally R. Partridge^1,*^, Steven P. Djordjevic^2,*,†^

^1^Centre for Infectious Diseases and Microbiology, The Westmead Institute for Medical Research, The University of Sydney and Westmead Hospital, Sydney, NSW, Australia

^2^The ithree Institute, University of Technology Sydney, Sydney, NSW, Australia

^*^These authors contributed equally to this work.

**
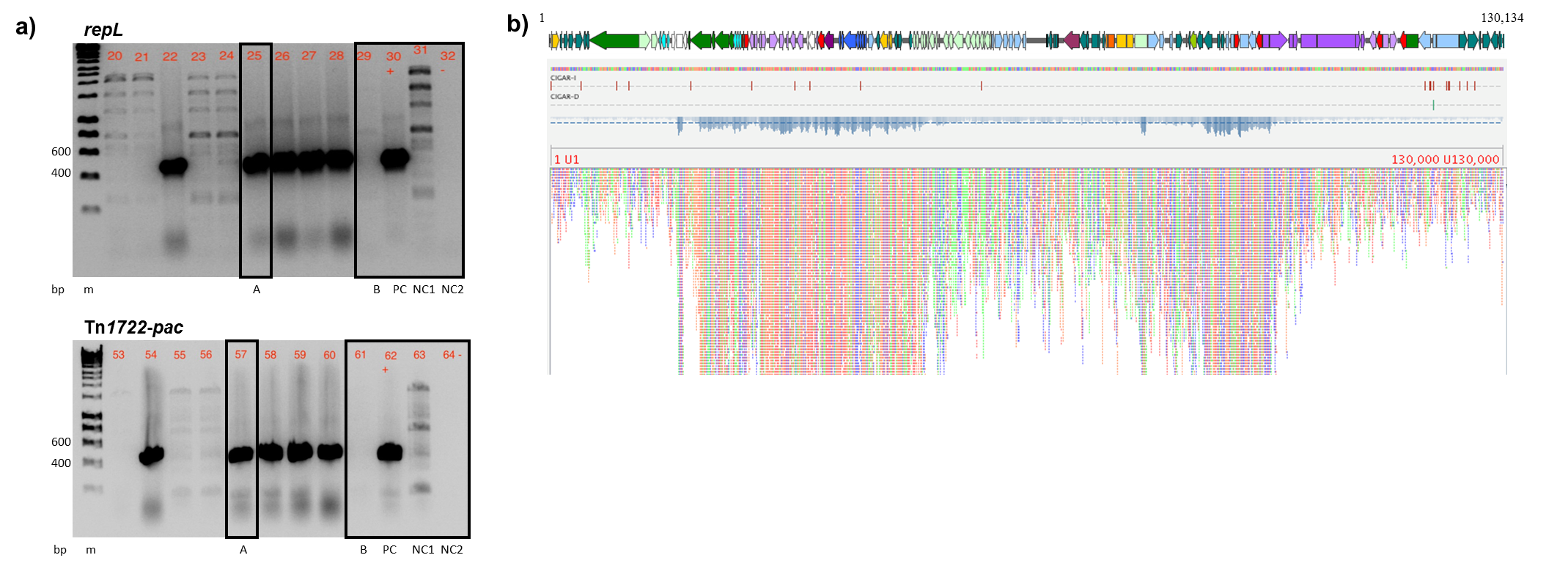
**

**Supplementary Figure 1. Detection of pTZ20_1P in lysogenized commensal *E. coli* WH17. a) Confirmatory PCRs after lysogenization.** *repL*, lytic replication gene, expected amplicon size 489 bp; Tn*1722*/pac, region spanning Tn*1722* (plasmid insertion) and *pacA* (phage P1), expected amplicon size 428 bp. Lanes highlighted by black boxes show: A (lanes 25, 57), lysogenized *E. coli* WH17 after co-incubation on solid medium with filtrate from *E. coli* TZ20_1P induced with mitomycin C (0.2 μg/mL); B (lanes 29, 61), *E. coli* WH17 co-incubated with filtrate from uninduced *E. coli* TZ20_1P; PC (lanes 30, 62), positive control *E. coli* TZ20_1P donor; NC1 (lanes 31, 63), negative control, *E. coli* WH17 recipient; NC2 (lanes 32, 64), negative control, molecular grade DNAse-free water. Additional lanes are from lysogenization of WH17 with: 20-21, mitomycin C in liquid medium; 22-25, mitomycin C on solid medium; 26-28, UV light (26-28) on solid medium; 53, UV light in liquid medium; 54-57, mitomycin C on solid medium; 58-60, UV light on solid medium. bp, base pairs; m, Hyperladder™ 1 kb Bioline (Meridian Bioscience). **b)** **Short-read mapping of *E. coli* WH17 lysogenized with pTZ20_1P** (lane A in gels)**.** Illumina raw reads aligned to the full length of the pTZ20_1P genome without gaps. Alignment mapped using Bowtie2^56^ v2.3.0 and visualized using Tablet^58^ v1.19.05.28.
